# Thrombectomy for acute large vessel occlusion in posterior and anterior circulation: a single institutional retrospective observational study

**DOI:** 10.1007/s00234-021-02799-4

**Published:** 2021-09-03

**Authors:** Tomoyoshi Kuribara, Satoshi Iihoshi, Eisuke Tsukagoshi, Akio Teranishi, Yu Kinoshita, Shin Sugasawa, Shinya Kohyama, Shinichi Takahashi, Hiroki Kurita

**Affiliations:** 1 Department of Endovascular Neurosurgery, Saitama Medical University International Medical Center, 1397-1 Yamane, Hidaka, Saitama 350-1298 Japan; 2 Department of Neurology and Cerebrovascular Medicine, Saitama Medical University International Medical Center, Hidaka, Saitama Japan; 3 Department of Cerebrovascular Surgery, Saitama Medical University International Medical Center, Hidaka, Saitama Japan

**Keywords:** Thrombectomy, Acute large vessel occlusion, Posterior circulation, Anterior circulation

## Abstract

**Purpose:**

Thrombectomy has been the gold standard therapy for anterior circulation occlusion; however, studies regarding thrombectomy in posterior circulation are lacking. In this study, we compared the efficiency of thrombectomy for acute large vessel occlusion between the posterior and anterior circulation at a single institution.

**Methods:**

We retrospectively analyzed consecutive patients who underwent thrombectomy for acute large vessel occlusion at our institution between August 2014 and April 2021. Differences in the clinical background, time course, and treatment technique and outcomes were evaluated between anterior and posterior circulation occlusions.

**Results:**

Overall, 353 patients (225 men and 128 women) were included: 314 patients had anterior circulation occlusion and 39 patients had posterior circulation occlusion. Between the patients with anterior and posterior circulation occlusions, the National Institutes of Health Stroke Scale (NIHSS) score (16 [12–21] vs. 29 [19–34], respectively, *p* < 0.001), door-to-puncture time (65 [45–99] vs. 99 [51–121] min, respectively, *p* = 0.018), and mortality (22 [7%] vs. 8 [20.5%] patients, respectively, *p* = 0.010) were significantly different; however, favorable outcome was not significantly different.

**Conclusion:**

Higher NIHSS score, delayed treatment, and higher mortality were observed in posterior circulation occlusion than in anterior circulation occlusion; successful reperfusion and favorable outcomes were similar between them. Similar favorable outcomes and reperfusion ratio to the anterior circulation might be achieved also in the posterior circulation; however, delayed treatment and the optimal first-pass strategy might need further improvement.

## Introduction

The effectiveness of thrombectomy using conventional devices for acute large vessel occlusion was lacking evidence for a long time [1–3]. However, this procedure has been accepted as the gold standard therapy based on evidence from five randomized controlled trials in which a stent retriever was used as the main device [4–8]. Additionally, a meta-analysis of these studies and two subsequent randomized controlled trials also confirmed the effectiveness of thrombectomy [9–11]. Subsequently, studies shifted their focus toward expanding the indications of eligible patients and improving the revascularization rate. The aforementioned studies reported the effectiveness of the procedure within 6 h from onset; however, two subsequent randomized controlled trials reported its effectiveness even after 6 h from onset in cases of mismatch between the clinical and imaging findings [12, 13]. Furthermore, the contact aspiration technique using large-bore catheters has been reported to be as effective as the stent retriever [14]; their combination is expected to improve the revascularization rate [15–18]. However, these studies focused on acute large vessel occlusion in the anterior circulation, and similar evidence in the posterior circulation remains lacking.

Posterior circulation occlusion, especially basilar artery (BA) occlusion, constitutes approximately 5% of large vessel occlusions [19, 20]. It is reported to result in a higher National Institutes of Health Stroke Scale (NIHSS) score, higher mortality, and lower rate of favorable outcomes in comparison with anterior circulation occlusion [21–25]. Despite the relatively high successful reperfusion rate, worse outcomes have been reported in prospective registry studies [26, 27]. A recent randomized controlled trial has reported no superiority of thrombectomy over the standard medical treatment within 8 h from onset [28]. Therefore, the optimal treatment strategy remains controversial. In contrast, a higher posterior circulation Alberta Stroke Program Early Computed Tomography Score (pc-ASPECTS), collateral status, shorter onset-to-reperfusion time, lower NIHSS score, higher Glasgow Coma Scale sum score, and lower baseline glycemic level have been reported to be associated with better outcomes [24, 29–31]. Therefore, better understanding of this disease might result in better outcomes. In this study, we evaluated and compared the characteristics of posterior circulation occlusion with those of anterior circulation occlusion at a single institution.

## Materials and methods

### Patients

This study protocol was approved by the ethics committee of our institution, and all patients provided informed consent with an opt-out policy. Between August 2014 and April 2021, consecutive patients who underwent thrombectomy for acute large vessel occlusion were enrolled in this study. In patients who underwent thrombectomy more than once during the study period at our institution, only the first procedure was evaluated. At our institution, the indications for thrombectomy included (1) acute large vessel occlusion (internal carotid artery [ICA]; the first segment of the middle cerebral artery [MCA], M1; the second segment of the MCA, M2; BA; vertebral artery [VA]) within 6 h from onset; (2) NIHSS score [32] ≥ 6; (3) DWI-ASPECTS [33] ≥ 6 in ICA and M1 occlusion [9]; (4) mismatch area ≥ 1 in DWI-ASPECTS between hyperintensity on diffusion-weighted imaging (DWI) and estimated perfusion area of the occluded vessel on magnetic resonance imaging (MRI) and magnetic resonance angiography (MRA) in M2 occlusion; (5) no pc-ASPECTS [34] based on DWI limit; and (6) no age limit. Patients with pre-onset modified Rankin Scale (mRS) [35] score ≥ 3 or early ischemic core ≤ 5 in DWI-ASPECTS were excluded. Additionally, recombinant tissue-type plasminogen activator (rt-PA) was intravenously administered to patients who had no contraindications within 4.5 h from onset. The MRI first policy is used for patients with suspected stroke using the 1.5-T or 3.0-T MRI system at our institution. Despite varied evaluation equipment, DWI, MRA, fluid-attenuated inversion recovery imaging, and T2- or T2*-weighted imaging have been routinely performed. In cases of inter-hospital transfer, we used the former hospital’s MRI and MRA evaluation as references. In MRA evaluation, the perfusion area of occluded vessels was assumed based on the location of these vessels, and no specific perfusion image evaluation was performed.

For patients with contraindications to MRI, non-contrast computed tomography (CT) or CT angiography was performed. Differences in clinical background, time course, and treatment outcomes were retrospectively evaluated between anterior and posterior circulation occlusions.

### Thrombectomy

The treatment strategy depended on several factors, such as the neuroendovascular surgeons, periods (differences in treatment devices and evidence at the time of treatment), location of lesion, and access routes. However, generally, all procedures were performed using biplane digital subtraction angiography equipment under sedation. For patients who needed endotracheal intubation due to impaired consciousness, general anesthesia was administered. The transfemoral approach was used, and the guiding catheter was navigated to the proximal portion of the occluded vessel along with an inner catheter and guidewire. Subsequently, the microcatheter was navigated to the distal portion of the occluded vessel with a microguidewire, and first-pass devices (stent alone, aspiration catheter alone, combined stent retriever with aspiration catheter, or other devices: percutaneous transluminal angioplasty [PTA] balloon, microguidewire and microcatheter, balloon guiding catheter [for suction], and stent) were used. The devices were removed during manual aspiration through the guiding catheter. This process was repeated until successful reperfusion was achieved. Additionally, angioplasty, stent placement, and medical therapies (other than rt-PA) were performed for residual spastic, atherosclerotic, or dissociative lesions. The degree of reperfusion was evaluated using the Thrombolysis in Cerebral Ischemia (TICI) scale [36]; TICI grade ≥ 2B was defined as successful reperfusion. In cases with difficulties in reperfusion, we terminated the procedure based on the onset time, perfusion area of the occluded vessel, and benefits of reperfusion after four passes. Treatment outcome was evaluated using mRS at discharge, and mRS ≤ 2 was defined as a favorable outcome.

### Statistical analysis

Data are expressed as median (interquartile range [IQR]). The Mann–Whitney *U* test, Fisher’s exact test, and Pearson’s chi-square test were used to compare the anterior and posterior circulation occlusions. Statistical analyses were performed using SPSS v26 (IBM Corp., Armonk, NY, USA), and *p* values < 0.05 indicated statistical significance. About the background characteristics, such as age, sex, comorbidity, history of smoking, inter-hospital transfer, drip-and-ship, in-hospital onset, NIHSS score, left-sided lesion, location of lesion, DWI-ASPECTS, pc-ASPECTS based on DWI, disease subtype, and tandem lesions, were evaluated. Regarding the treatment and outcome, intravenous rt-PA, first-pass devices (the devices used in the first passes), PTA, stent placement, medical therapy (other than rt-PA), time course, TICI 2B-3 reperfusion, TICI 3 reperfusion, first-pass TICI 2B-3 reperfusion, symptomatic complications, hospitalization period, mRS at discharge, favorable outcomes, and mortality were evaluated.

## Results

### Background characteristics

A total of 353 patients (225 men and 128 women) were included in the study. The median age was 76 (69–82) years (range, 32–96). A total of 314 patients had anterior circulation occlusion, and 39 patients had posterior circulation occlusion. The background data of patients with anterior and posterior circulation occlusions are summarized in Table 1. The locations of the lesions included the ICA (*n* = 120); M1 (*n* = 185); M2 (*n* = 43); BA (*n* = 37); VA (*n* = 6); tandem lesions (*n* = 37); and bilateral lesions (*n* = 1). Between the patients with anterior and posterior circulation occlusions, the NIHSS score (16 [12–21] vs. 29 [19–34], respectively, *p* < 0.001) and intracranial stenotic occlusions (26 [8.3%] vs. 9 [23.1%] patients, respectively, *p* = 0.008) were significantly different; however, other characteristics (age, sex, comorbidities, history of smoking, inter-hospital transfer, drip-and-ship, in-hospital onset, left-sided lesion, preprocedural MRI evaluation, location of lesion, DWI-ASPECTS, pc-ASPECTS based on DWI, disease subtype, tandem lesion, and extracranial stenotic occlusion) demonstrated no significant differences.

**Table 1:** Background data of patients with anterior and posterior circulation occlusion

Characteristics	Total	Anterior circulation occlusion	Posterior circulation occlusion	*p* value
Number of patients	353	314	39	
Age, median (IQR), y	76 (69–82)	76 (69–82)	74 (69–79.5)	0.310
Sex, male (%)	225 (63.7)	197 (62.7)	28 (71.8)	0.267
Coexisting disease				
Hypertension (%)	209 (59.2)	186 (59.2)	23 (59.0)	0.975
Diabetes mellitus (%)	80 (22.7)	71 (22.6)	9 (23.1)	0.948
Hyperlipidemia (%)	98 (27.8)	90 (28.7)	8 (20.5)	0.284
Atrial fibrillation (%)	200 (56.7)	181 (57.6)	19 (48.7)	0.289
Coronary artery disease (%)	48 (13.6)	39 (12.4)	9 (23.1)	0.067
Previous smoking (%)	120 (34.0)	109 (34.7)	11 (28.2)	0.418
Inter-hospital transfer (%)	83 (23.5)	70 (22.3)	13 (33.3)	0.125
Drip-and-ship (%)	24 (6.8)	19 (6.1)	5 (12.8)	0.165
In-hospital onset (%)	25 (7.1)	23 (7.3)	2 (5.1)	1.000
NIHSS score, median (IQR)	17 (12–23)	16 (12–21)	29 (19–34)	< 0.001*
Left-sided lesion (%)	150 (42.5)	148 (47.1)	2 (5.1)	
Location of lesion				
ICA (%)		120 (38.2)		
M1 (%)		185 (58.9)		
M2 (%)		43 (13.7)		
BA (%)			37 (94.9)	
VA (%)			6 (15.4)	
ASPECTS, median (IQR)		8 (6–9)		
pc-ASPECTS, median (IQR)			7 (6–8.5)	
Disease subtype				
Cardiogenic (%)	212 (60.1)	193 (61.5)	19 (48.7)	0.125
Atherosclerotic (%)	76 (21.5)	65 (20.7)	11 (28.2)	0.282
Others (%)	65 (18.4)	56 (17.8)	9 (23.1)	0.426
Tandem lesion	37 (10.5)	33 (10.5)	4 (10.3)	1.000
Intracranial stenotic lesion (%)	35 (9.9)	26 (8.3)	9 (23.1)	0.008*
Extracranial stenotic lesion (%)	41 (11.6)	39 (12.4)	2 (5.1)	0.286

^*^p < 0.05. *IQR* interquartile range, *NIHSS* Institutes of Health Stroke Scale, *ICA* internal carotid artery, *M1* first segment of middle cerebral artery, *M2* second segment of middle cerebral artery, *BA* basilar artery, *VA* vertebral artery, *ASPECTS* Alberta Stroke Program Early Computed Tomography Score, *pc* posterior circulation

### Treatment, time course, and outcome

**Fig. 1:**
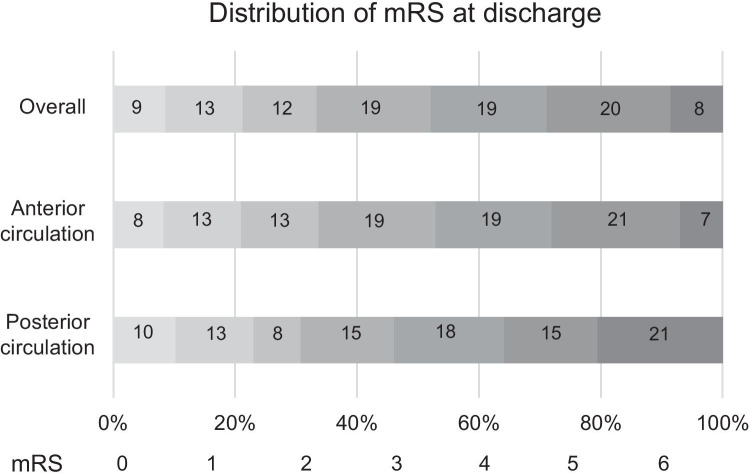
Distribution of the modified Rankin Scale (mRS) scores at discharge in all patients and in those with anterior and posterior circulation occlusions

The treatment, time course, and outcomes are summarized in Table 2. Intravenous rt-PA was administered to 180 (51.0%) patients. The first-pass devices included the stent retriever in 101 (28.6%) patients, aspiration catheter in 123 (34.8%) patients, combined approach in 103 (29.2%) patients, and other devices in 26 (7.4%) patients (PTA [*n* = 14], microguidewire and microcatheter [*n* = 2], suction through a balloon guiding catheter [*n* = 4], stent placement [*n* = 1], and inaccessible [*n* = 5]). PTA was performed in 46 (13.0%) patients, stent placement in 19 (5.4%) patients, and medical therapy other than rt-PA in 35 (9.9%) patients. Stent placement was performed for both intracranial and extracranial steno-occlusive lesions (cervical ICA [*n* = 15], M1 [*n* = 2], BA [*n* = 1], and extracranial VA [*n* = 1]). The details of the stents and location were as follows: Carotid Wallstent (Boston Scientific, Natick, MA, USA) (cervical ICA [*n* = 12]), Integrity bare metal stent (Medtronic, Minneapolis, MN, USA) (M1 [*n* = 1], BA [*n* = 1], and extracranial VA [*n* = 1]), Enterprise 2 VRD (Codman Neurovascular, Johnson & Johnson, Miami, FL, USA) (cervical ICA [*n* = 2]), the Wingspan Stent System (Stryker, Kalamazoo, MI, USA) (M1 [*n* = 1]), and CASPER stent (Microvention, Terumo, Tustin, CA, USA) (cervical ICA [*n* = 1]). Between the patients with anterior and posterior circulation occlusions, the door-to-picture time (17 [12–24] vs. 24 [16–36] min, respectively, *p* = 0.005), door-to-needle time (72 [61–85] vs. 93 [83–123] min, respectively, *p* < 0.001), door-to-puncture time (65 [45–99] vs. 99 [51–121] min, respectively, *p* = 0.018), door-to-reperfusion time (125 [90–156] vs. 163 [121–203] min, respectively, *p* = 0.004), the number of passes in the cases that were not achieved successful reperfusion in the first pass (3[ 2–4] vs. 2 [2, 3], respectively, *p* = 0.036), and mortality (22 [7%] vs. 8 [20.5%] patients, respectively, *p* = 0.010) were significantly different. However, other characteristics, such as intravenous rt-PA, first-pass devices, PTA, stent placement, medical therapy other than rt-PA, onset-to-door time, puncture-to-reperfusion time, onset-to-reperfusion time, TICI 3 reperfusion, first-pass TICI 2B-3 reperfusion, symptomatic complications, and mRS at discharge, demonstrated no significant differences between them. Particularly, TICI 2B-3 reperfusion (257 [81.8%] vs. 30 [76.9%] patients, respectively, *p* = 0.457) and favorable outcome (106 [33.8%] vs. 12 [30.8%] patients, respectively, *p* = 0.709) were not significantly different between the patients. The details of mRS at discharge in all patients and in those with anterior and posterior circulation occlusions are summarized in Fig. 1.

**Table 2:** Treatment, time course, and outcomes in patients with anterior and posterior circulation occlusion

Characteristics	Total	Anterior circulation occlusion	Posterior circulation occlusion	*p* value
Intravenous rt-PA (%)	180 (51.0)	162 (51.6)	18 (46.2)	0.522
First-pass devices				
Stent retriever (%)	101 (28.6)	90 (28.7)	11 (28.2)	0.952
Aspiration catheter (%)	123 (34.8)	110 (35.0)	13 (33.3)	0.834
Combined approach (%)	103 (29.2)	93 (29.6)	10 (25.6)	0.606
Other devices (%)	26 (7.4)	21 (6.7)	5 (12.8)	0.187
PTA (%)	46 (13.0)	41 (13.1)	5 (12.8)	0.967
Stent placement (%)	19 (5.4)	17 (5.4)	2 (5.1)	1.000
Medical therapy other than rt-PA (%)	35 (9.9)	34 10.8)	1 (2.6)	0.152
Time course				
Onset-to-door time, median (IQR), min	112.5 (58.8–240.5)	112 (60–241)	117 (53–314)	0.888
Door-to-picture time, median (IQR), min	17 (12–25)	17 (12–24)	24 (16–36)	0.005*
Door-to-needle time, median (IQR), min	74 (61–89)	72 (61–85)	93 (83–123)	< 0.001*
Door-to-puncture time, median (IQR), min	69 (46–102)	65 (45–99)	99 (51–121)	0.018*
Picture to puncture time, median (IQR), min	56.5 (36–80.3)	52 (35–76.5)	77 (60–96)	0.001*
Puncture-to-reperfusion time, median (IQR), min	49 (31–74)	48 (31–74)	52 (37–77)	0.384
Door-to-reperfusion time, median (IQR), min	127 (93–165)	125 (90–156)	163 (121–203)	0.004*
Onset-to-reperfusion time, median (IQR), min	270 (195–376)	267 (191–370)	295 (231–442)	0.079
TICI 2B-3 reperfusion (%)	287 (81.3)	257 (81.8)	30 (76.9)	0.457
TICI 3 reperfusion (%)	160 (45.3)	141 (44.9)	19 (48.7)	0.652
First-pass TICI 2B-3 reperfusion (%)	157 (44.4)	137 (43.6)	20 (51.3)	0.364
Number of passes^†^, median (IQR)	3 (2–4)	3 (2–4)	2 (2–3)	0.036*
Symptomatic complication				
Ischemic complication (%)	2 (0.6)	2 (0.6)	0 (0)	1.000
Hemorrhagic complication (%)	20 (5.7)	19 (6.1)	1 (2.6)	0.711
Puncture related complication (%)	8 (2.3)	7 (2.2)	1 (2.6)	1.000
Hospitalization period (IQR), day	29 (18–41)	29.5 (18.3–41)	27 (12–39)	0.226
mRS at discharge	3 (2–5)	3 (2–5)	4 (2–5)	0.325
mRS ≤ 2 at discharge (%)	118 (33.4)	106 (33.8)	12 (30.8)	0.709
Mortality (%)	30 (8.5)	22 (7.0)	8 (20.5)	0.010*

^*^p < 0.05. †In cases that were not achieved successful reperfusion in the first pass

*rt-PA* recombinant tissue-type plasminogen activator, *PTA* percutaneous transluminal angioplasty, *IQR* interquartile range, *TICI* Thrombolysis in Cerebral Ischemia

In addition, subgroup analysis according to the first-pass device was performed, and puncture-to-reperfusion time, rate of TICI 2B-3 reperfusion, TICI 3 reperfusion, first-pass TICI 2B-3 reperfusion, symptomatic complications, favorable outcome, and mortality due to first-pass devices were evaluated (Table 3). In those with anterior circulation occlusion, a longer puncture-to-reperfusion time was observed with the combined approach than that with the aspiration catheter alone (53.5 [36.5–77] vs. 41.5 [28–69] min, respectively, *p* = 0.045); however, there was no significant difference between other devices and posterior circulation occlusion. In patients with posterior circulation occlusion, a higher rate of first-pass TICI 2B-3 reperfusion was observed with stent retrievers than that with aspiration catheters (9 [81.8%] vs. 5 [38.5%] patients, respectively, *p* = 0.047); however, there was no significant difference among other devices and anterior circulation occlusion. A higher rate of hemorrhagic complications was observed with the combined approach than with the aspiration catheter (3 [2.7%] vs. 10 [10.8%] patients, respectively, *p* = 0.020) in anterior circulation, while there was no significant difference among other devices and anterior circulation occlusion. In addition, BA stenotic occlusions were seen in 2 (18.2%), 2 (15.4%), 3 (30.0%), and 2 (40%) patients who underwent thrombectomy with stent retriever, aspiration catheter, combined approach, and other devices as first-pass devices for posterior circulation occlusion, respectively.

**Table 3:** Subgroup analysis according to the first-pass device

Characteristics	Location	First-pass device			*p* value		
		1. Stent retriever	2. Aspiration catheter	3. Combined approach	1 vs. 2	1 vs. 3	2 vs. 3
Number of patients	AC	90	110	93			
	PC	11	13	10			
Puncture-to-reperfusion time, median (IQR), min	AC	47 (28.3–64.8)	41.5 (28–69)	53.5 (36.5–77)	0.993	0.065	0.045*
	PC	54 (43–65)	38.5 (26–79.3)	48 (42–74)	0.566	0.824	0.382
TICI 2B-3 reperfusion (%)	AC	75 (83.3)	91 (82.7)	77 (82.8)	0.910	0.923	0.990
	PC	10 (90.9)	9 (69.2)	8 (80.0)	0.327	0.586	0.660
TICI 3 reperfusion (%)	AC	39 (43.4)	57 (51.8)	36 (38.7)	0.232	0.525	0.062
	PC	7 (63.6)	5 (38.5)	7 (70.0)	0.219	1.000	0.214
First-pass TICI 2B-3 reperfusion (%)	AC	41 (45.6)	48 (43.6)	44 (47.3)	0.786	0.812	0.600
	PC	9 (81.8)	5 (38.5)	5 (50.0)	0.047*	0.183	0.685
Symptomatic ischemic complications (%)	AC	0 (0)	1 (9.1)	0 (0)	1.000	NA	1.000
	PC	0 (0)	0 (0)	0 (0)	NA	NA	NA
Symptomatic hemorrhagic complications (%)	AC	3 (3.3)	3 (2.7)	10 (10.8)	0.803	0.051	0.020*
	PC	0 (0)	1 (7.7)	0 (0)	1.000	NA	1.000
mRS ≤ 2 at discharge (%)	AC	30 (33.3)	43 (39.1)	26 (28.0)	0.400	0.430	0.095
	PC	4 (36.4)	4 (30.8)	2 (20.0)	1.000	0.635	0.660
Mortality (%)	AC	4 (4.4)	8 (7.3)	7 (7.5)	0.402	0.380	0.945
	PC	1 (9.1)	3 (23.1)	2 (20.0)	0.596	0.586	1.000

^*^p < 0.05. *IQR* interquartile range, *AC* anterior circulation, *PC* posterior circulation, *TICI* Thrombolysis in Cerebral Ischemia, *mRS*, modified Rankin Scale

## Discussion

### Differences between anterior and posterior circulation occlusions

In this study, we evaluated the characteristics of patients with posterior circulation occlusion and compared them with those with anterior circulation occlusion. Higher NIHSS; more intracranial stenotic lesions; longer door-to-picture time, door-to-needle time, door-to-puncture time, and door-to-reperfusion time; fewer passes; and higher mortality were significantly different, while successful reperfusion and favorable outcome demonstrated no significant differences between the groups. Previous reports have demonstrated that male preponderance [24], younger age [24, 37], higher NIHSS score [21, 22, 24], longer onset-to-puncture time [21, 37], longer onset-to-reperfusion time [37], higher rate of futile reperfusion [25], lower tendency of favorable outcome [21–23, 25], and higher mortality [22, 23, 25] were observed in patients with posterior circulation occlusion. In contrast, there have also been some studies that reported similar or lower NIHSS scores [37, 38], lower rates of symptomatic hemorrhage [25, 37], similar rates of favorable outcome [37, 38], and similar mortality [24, 37, 38] between patients with anterior and those with posterior circulation occlusions. Our findings confirmed these characteristics using real-world clinical data from a single institution.

Posterior circulation is densely surrounded by areas responsible for sensory and motor functions as well as maintaining awake conditions; therefore, patients with posterior circulation occlusion tend to present in a severe state. Consequently, the NIHSS score in these patients could be higher than that in patients with anterior circulation occlusion. Additionally, the NIHSS score tended to be higher in patients with impaired consciousness, which may have affected the difference. In contrast, some studies have reported similar or lower NIHSS scores in posterior circulation occlusion in comparison with anterior circulation occlusion. These reports only included patients who underwent thrombectomy. However, because of the severity of presentation, patients with posterior circulation occlusion often miss the opportunity for acute recanalization therapy [39]; therefore, the NIHSS score might be underestimated. Additionally, the posterior circulation system is more resistant to ischemic insult with slower progression due to the greater amount of white matter and better collateral circulation than the anterior system [40, 41]. These features might have resulted in the lower NIHSS score in some patients. About disease subtype, in our study, patients with posterior circulation occlusion had more intracranial stenotic occlusions, although the successful reperfusion ratio did not significantly differ. In previous reports, higher rates of intracranial atherosclerosis-related occlusions were observed in patients with posterior circulation occlusion than in those with anterior circulation occlusion, and successful reperfusion rates were not significantly different between atherosclerosis-related and non-atherosclerosis-related occlusions [42]. Our study also confirmed these findings. Patients with posterior circulation occlusion need more time to be stabilized in the emergency room, such as the time required for endotracheal intubation, due to impaired consciousness. Additionally, a previous report has demonstrated that symptoms considered typical of posterior circulation infarction occur far less often than expected [43]. Various presentations were observed with posterior circulation occlusion in comparison with those with anterior circulation. We sometimes are not aware of the possibility of large vessel occlusion before imaging has been performed. These factors might lead to a delay in diagnosis as well as delay of treatment as observed in our results in posterior circulation occlusion. The number of passes in the cases that were not achieved successful reperfusion in the first pass, smaller diameter of vessels, and concerns of vascular damage might explain the fewer passes in the posterior circulation. From the perspective of outcome, a lower tendency of favorable outcome and higher mortality in posterior circulation occlusion might be due to the severity, such as higher NIHSS score. In prospective registry studies, despite the relatively high successful reperfusion rate, worse outcomes have been reported in posterior circulation occlusion [26, 27]. In contrast, despite the delayed treatment, some reports and our results demonstrate similar favorable outcomes and successful reperfusion ratio in posterior circulation occlusion as those in anterior circulation occlusion [24, 37, 38]. The aforementioned stronger resistance to ischemic insult might have affected these results. In our study, compared with patients with anterior circulation occlusion, a higher NIHSS score, more intracranial stenotic occlusions, delayed treatment, fewer passes, and higher mortality, as well as similar successful reperfusion rates and favorable outcomes, were observed in patients with posterior circulation occlusion. Similar favorable outcomes might be due to the similar successful reperfusion ratio, and improvements regarding delayed treatment and optimal first-pass strategy might result in better outcomes.

### Optimal treatment strategy

Regarding the treatment strategy for posterior circulation occlusion, although there were only 39 patients, stent retrievers resulted in a higher rate of first-pass reperfusion with similar puncture-to-reperfusion time, rate of symptomatic complications, tendency of favorable outcome, and mortality as those with aspiration catheter and the combined approach in our subgroup analysis according to first-pass device. In a retrospective multicenter international study on posterior circulation occlusion, significantly shorter procedure time, higher TICI score, and higher tendency of favorable outcome were observed with the direct aspiration first-pass (ADAPT) technique in comparison with stent retrievers; however, there was no significant difference in comparison with the combined approach [21]. Additionally, Gory et al. reported that the ADAPT technique as the first-line strategy for posterior circulation occlusion resulted in a higher rate of TICI 3 reperfusion and shorter procedure [44]. These reports suggest that stent retriever could require more procedure time and lead to a lesser than favorable outcome. In contrast, Maus et al. reported that stent retrievers combined with aspiration catheters (stent retriever-assisted vacuum-locked extraction technique) resulted in a higher reperfusion rate than aspiration catheter only [45]. Therefore, aspiration or a combined approach might be recommended as a first-pass device for posterior circulation occlusion. However, in posterior circulation occlusion, the vessel diameter is sometimes small and the treatment options might be limited. A stent retriever can be used through a relatively small guiding catheter in these cases and may be considered the first choice based on our results.

### Limitations and future work

This study had several limitations. The first treatment strategy depended on the neuroendovascular surgeons, periods, locations of lesions, and access routes, which might have affected the results. Second, the clinical outcome was based on the patient condition at discharge, and the median hospitalization period was only 29 days (range, 18–41). Most studies are based on the patient’s condition at 90 days after the procedure; therefore, this difference might have affected our results. Finally, the retrospective study design and inadequate sample size might have weakened our findings. In the near future, it will be necessary to confirm our results in prospective studies with large sample sizes.

## Conclusions

In this study, we compared the efficiency of thrombectomy for acute large vessel occlusion between the posterior and anterior circulation at a single institution. In comparison with patients with anterior circulation occlusion, those with posterior circulation had a significantly higher NIHSS score, more intracranial stenotic occlusions, delayed treatment, fewer passes, and higher mortality, while successful reperfusion and favorable outcomes were not significantly different between the two groups. Additionally, although the number of patients was small, the stent-first strategy resulted in a higher rate of first-pass reperfusion with similar puncture-to-reperfusion time, rate of symptomatic complications, tendency of favorable outcome, and mortality as other strategies and might be considered a first-pass device for posterior circulation occlusion depending on the vessel diameter. Similar favorable outcomes and reperfusion ratio to the anterior circulation might be achieved also in the posterior circulation; however, delayed treatment and the optimal first-pass strategy might need further improvement.
